# NucliTrack: an integrated nuclei tracking application

**DOI:** 10.1093/bioinformatics/btx404

**Published:** 2017-06-20

**Authors:** Sam Cooper, Alexis R Barr, Robert Glen, Chris Bakal

**Affiliations:** 1 Division of Cancer Biology, The Institute of Cancer Research, London, UK; 2 Department of Computational Systems Medicine, Imperial College, South Kensington Campus, London, UK

## Abstract

**Summary:**

Live imaging studies give unparalleled insight into dynamic single cell behaviours and fate decisions. However, the challenge of reliably tracking single cells over long periods of time limits both the throughput and ease with which such studies can be performed. Here, we present NucliTrack, a cross platform solution for automatically segmenting, tracking and extracting features from fluorescently labelled nuclei. NucliTrack performs similarly to other state-of-the-art cell tracking algorithms, but NucliTrack’s interactive, graphical interface makes it significantly more user friendly.

**Availability and implementation:**

NucliTrack is available as a free, cross platform application and open source Python package. Installation details and documentation are at: http://nuclitrack.readthedocs.io/en/latest/ A video guide can be viewed online: https://www.youtube.com/watch?v=J6e0D9F-qSU Source code is available through Github: https://github.com/samocooper/nuclitrack. A Matlab toolbox is also available at: https://uk.mathworks.com/matlabcentral/fileexchange/61479-samocooper-nuclitrack-matlab.

**Supplementary information:**

Supplementary data are available at *Bioinformatics* online.

## 1 Introduction

Live imaging studies are now allowing us to explore the previously shrouded world of mammalian cell signalling dynamics. For example, imaging populations of live single cells over several days has revealed how signalling dynamics can control key cell fate decisions (Cooper and Bakal, 2017). However, hindering the ease, time-periods and throughput with which live single-cell studies can be performed, are challenges in automatically tracking fluorescently labelled objects (cells, nuclei or organelles) accurately over time periods often exceeding several days, sometimes in highly motile cells (Cabantous *et al.*, 2005).

**Fig. 1:**
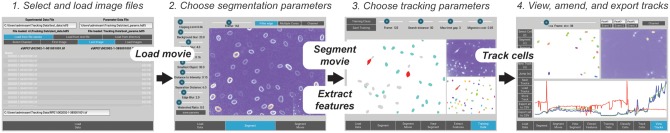
The Nuclitrack application workflow: This figure shows the four main user interface windows used to track nuclei in Nuclitrack: (1) *File loading*: In this interface the user can browse files and load the time series imaging data, as well as selecting file names for storing tracking data; (2) *Segmentation*: The sliders on this interface allow the user to interactively choose segmentation parameters for identifying cell nuclei; (3) *Training data selection*: As tracking uses a probabilistic algorithm, examples of single nuclei, nuclei about to enter and exit mitosis, as well as incorrectly segmented nuclei must be given; (4) *Track visualization, correction and export*: In this interface the user can explore the tracking results, and view tracking data in the graph window. Additionally functions exist to correct tracks. Finally, the user can export single cell data from either all tracks or a subset of selected tracks

To date, the majority of cell tracking software relies on ‘frame-to-frame linking’ of segmented nuclei or cells (Meijering *et al.*, 2012). In such methods, errors in segmentation, where, for example, either multiple nuclei are labelled as a single segment, or a nucleus is missed over several timeframes resulting in a tracking gap, can severely compromise tracking results. To handle such difficulties, global optimization methods have now been developed (Milan *et al.*, 2016). Yet, implementations of global optimization approaches to tracking in cell biology research have generally been limited to standalone packages, meaning that segmentation, track inspection and track correction have to be performed in other software which is non-trivial for scientists with little programming expertise (Hilsenbeck *et al.*, 2016). Therefore, we have developed NucliTrack an integrated Python package that allows efficient segmentation, feature extraction and tracking of fluorescently labelled nuclei or cells through an intuitive graphical interface (Fig. 1). Additionally, the interface also includes tools for inspecting, correcting and exporting data on tracked nuclei.

## 2 Implementation

The identification and segmentation of fluorescently labelled objects in NucliTrack is based on marker controlled watershed segmentation, built with the scikit image Python library (van der Walt *et al.*, 2014). We utilized a novel Python application development library ‘Kivy’ to create an interface that allows users to interactively vary segmentation parameters (Virbel *et al.*, 2011). Once effective parameters are chosen, segmentation is then performed on the entire imaging sequence.

In many types of image analysis workflows aimed at quantifying cellular phenotypes, the segmentation step can result in errors, which must be handled by tracking algorithms. Moreover, cells undergoing cell division must also be detected. To address this, we use a probabilistic approach described by Magnusson *et al.* to perform tracking (Magnusson *et al.*, 2015). This requires training data to be selected that contains examples of any erroneous segmentation, as well as normal, mitotic and post-mitotic cells. Training is performed within the user interface prior to tracking. The tracking algorithm then seeks to globally optimize the set of all tracks, by iteratively adding the highest scoring track given the current set of tracks. Tracks are scored with a cost function which penalizes large movements, gaps between frames, and passage through segments which are unlikely to be nuclei, whilst highly scoring tracks that minimize movement between frames, include few gaps and pass through segments likely to be nuclei. A dynamic programming technique is then used to find the highest scoring track that is added to the set of all tracks. Importantly, in identifying the highest scoring track, swaps with neighbouring tracks may be performed, thus minimizing the likelihood that a local, rather than a global, optimum is found.

After successful tracking of objects, tracks can be edited within the user interface. Here, three windows showing: (i) colour-coded tracked segments; (ii) the original video; and (iii) feature values over the full length of a selected track (in the form of a line plot over time), allow the user to easily analyze and inspect tracks for errors, or anomalous results. Moreover, using a set of tools with keyboard hotkeys the user can efficiently navigate the video, manually correct errors in tracking, and add flags to mark specific events, before exporting results. Throughout NucliTrack we have also incorporated data management steps, such that intermediate steps are saved in the HDF5 file format, through the Python h5py package (Collette, 2013). Parameters are stored in a separate HDF5 file meaning that the segmentation and training stages can be skipped when analyzing subsequent movies. A function also exists in the Python package that accommodates for batch processing of videos using the HDF5 parameter file.

To characterize the performance of the global optimization approach to automated cell tracking we tested NucliTrack against a benchmark set of fluorescently labelled nuclei videos (Maška *et al.*, 2014; Svoboda *et al.*, 2009). Here NucliTrack performed similarly to other global optimization approaches (Supplementary Methods). The performance of NucliTrack was limited by the more basic segmentation procedures we adopt, however we believe having an intuitive and interactive workflow is significantly more important for users with less technical expertise. Labeled images, segmented in alternative programs may also be imported in the file loading step.

NucliTrack is an easy to use package for tracking and extracting data from fluorescently labelled cells or nuclei, in significantly higher throughput than manual tracking can achieve. Importantly, by allowing users to quickly inspect and correct tracking data, near 100% accuracy can be achieved, which is critical in quantifying cell fate decisions. For example, NucliTrack allowed us to study how DNA damage influences the proliferation-quiescence decision in thousands of cells, tracked for hundreds of frames, over multiple days (Barr *et al.*, 2017). Finally, by coupling established packages for data processing in Python, with more recent solutions to data management (h5py), graphical user interface development (kivy) and image processing (skimage), this software also provides an example of how user friendly, scientific software may be efficiently developed in an open source environment.

## Funding

S.C. is supported by the NIHR Imperial BRC, and the STRATiGRAD graduate training program. C.B. and A.R.B. are funded by a BBSRC Strategic LoLa grant [BB/M00354X/1], C.B. is also funded by a Cancer Research UK Programme Foundation Award [C37275/1A20146]. 

*Conflict of Interest:* none declared.

## Supplementary Material

Supplementary Data
