# Supplementary material for: NucliTrack: an integrated nuclei tracking application
Source: Bioinformatics. 2017 Jun 20;33(20):3320–2. doi: 10.1093/bioinformatics/btx404 (PMC5860035; doi:10.1093/bioinformatics/btx404)
Supplement: Supplementary Data [file btx404_nuclitrack_supplemental.pdf]

Comparison of segmentation and tracking in NucliTrack

To test the performance of NucliTrack, we automatically segmented and tracked six different videos that have been made available as part of the the ISBI Cell Tracking Challenge (http://www.codesolorzano.com/Challenges/CTC/Datasets.html). Each video is a time series acquisition of fluorescently-labelled nuclei, from three labelled cell lines. These lines are: GFP-GOWT1 mouse stem cells, H2B-GFP HeLa cells, and simulated nuclei of HL60 cells stained with Hoescht. Exported tracks were then compared to ground truth values to obtain a tracking precision of the method score (TRA).

We compared the TRA values we obtained on the available training datasets to the TRA values obtained by the top teams on test data. Whilst the comparison is on training data versus test data, the results indicate that NucliTrack is able to obtain tracking results of a similar standard to other top performing algorithms.

**Competition Performance**			
Rank	N2DH-GOWT1	N2DL-HeLa	N2DH-SIM
1	0.976	0.991	0.975
2	0.925	0.986	0.957
3	0.916	0.982	0.948
**Performance on Training Datasets**			
NucliTrack	**0.952**	**0.943**	**0.97**

Teams:

KTH-SE	KIT-GE	CUNI-CZ	HD-Har-GE
HD-Hau-GE	FR-Ro-GE	PAST-FR	

A full description of team results can be found at:

http://www.codesolorzano.com/Challenges/CTC/Latest_Results.html
